# Improving health outcomes: innovation, coverage, quality and adherence

**DOI:** 10.1186/2045-4015-1-43

**Published:** 2012-10-25

**Authors:** Martin McKee, Clara K Chow

**Affiliations:** 1 European Centre on Health of Societies in Transition, London School of Hygiene and Tropical Medicine, London, UK; 2 The George Institute for Global Health and Sydney Medical School, University of Sydney, Sydney, Australia

## Abstract

The Israeli health system has made considerable progress in reducing deaths amenable to medical care but has more to do. This commentary describes how progress in this area results from innovation, coverage, quality, and adherence to treatment. It describes what is being done in Israel and beyond to address each of these factors but concentrates on the often poorly recognised problem of adherence to treatment, describing the growing evidence that it is often sub-optimal and reviewing evidence on what can be done to improve it.

## Introduction

In the accompanying paper, Goldberger and Haklai describe how the Israeli health system has made considerable progress in reducing deaths amenable to medical care in the past decade [1]. Deaths from these causes have fallen by slightly over 3% per annum, which is better than has been achieved in a number of European countries, but still not as good as the best performers. This raises the question of what else needs to be done to achieve even better results in the next decade.

Reductions in deaths amenable to medical care come about for four main reasons. The first is innovation, with new models of care being developed and implemented. Historically, examples include the discovery of penicillin as a means to treat common bacterial infections, the development of anti-retrovirals to treat HIV infection, and the use of chemotherapy to treat cancers such as childhood leukaemia and testicular cancer. More often, the effects are less dramatic, as new drugs incrementally improve outcome, such as the use of combination therapy to treat hypertension. However, innovation alone makes relatively little contribution to health outcomes; what matters is that the new approaches to health care reach those in need. This takes us to the second factor, coverage of the population by health care. The impact is most easily seen where health care is not provided universally, most notably in the United States, where rates of amenable mortality lag increasingly far behind those seen in European health systems [2]. However, even in Israel, where coverage is universal, the high level of co-payments and other costs of obtaining care mean that a substantially higher proportion of the population forego treatment than in comparable European countries. [3] The third factor is quality of care. Improvements in the outcomes from major trauma in British emergency departments were not due to innovative treatments or to improved access to care, given the existence of the National Health Service. What was found to be important was a combination of many small changes, such as improved training of staff in advanced life support, greater involvement of senior doctors, and the implementation of quality assurance systems [4]. However, there is a fourth, which tends to be rather less well recognised but is likely to be at least as important. This is adherence to treatment, something which is often much less than health professionals and policymakers realise.

### What are the priorities?

Which of these should Israeli policy makers prioritise to improve Israel's relative position with regard to overall amenable mortality and the pattern of cause-specific amenable mortality Therapeutic innovation has long ago moved into the global arena and while there may be economic arguments for strengthening the Israeli pharmaceutical industry, it is not especially important for population health. Population coverage should not be a problem, given the provision of universal coverage, although as Goldberger and Haklai note, coverage is uneven, both geographically and by ethnicity. There is always more that can be done to improve quality, especially in a health system that is under increasing financial constraints that place pressures on staff [5], but Israel has established functioning quality assurance systems that attract widespread professional support [6,7] and achieves good performance on process measures [7]. There has, however, been rather less attention to adherence to prescribed therapy, even though the limited research that has been done shows high levels of non-adherence [8]. Thus, while all of these aspects of care require attention, and in particular the evidence of foregone coverage due to costs of care due to co-payments, this commentary focuses on only one element, adherence to treatment, while noting that efforts to address this issue should be part of a comprehensive approach to achieving health gain.

The evidence from Israel is consistent with follow up data on patients elsewhere with common chronic conditions showing that up to 70% discontinue their prescribed treatment [9,10] even though health professionals are often unaware of it, [11] with one study finding that over 80% of patients would not tell their physicians that they were not taking their prescribed medicines [12]. Non-adherence is found among people of all sorts, with little impact of demographic variables, although some variation by condition being treated (e.g. higher adherence with HIV and cancer and lower with diabetes and pulmonary disease) [13].

Non-adherence matters. Blood pressure control is, unsurprisingly, substantially associated with adherence to medication [14]. One study, using data from a clinical trial, and thus including patients who one might expect to be more motivated than average, found that a third had discontinued at least some of the treatment prescribed (aspirin, beta-blockers, and statins) within 12 months following a myocardial infarction [15]. Those that discontinued treatment were almost four times as likely to die. Similar findings were reported from a French study [16]. A study in an American managed care organisation found that over 20% of those with diabetes took their medication (oral hypoglycemics, antihypertensives, and statins) on at least 80% of days. Those who did not take their medication regularly were significantly more likely to be hospitalised or die [17]. A similar study of women prescribed adjuvant hormonal treatment for breast cancer found that 31% discontinued therapy and, of the remainder, 28% did not adhere regularly to it. Those who discontinued therapy were 26% more likely to die (95% confidence intervals 9-46%) while those not adhering were 49% more likely (95% confidence intervals 23-81%) [18]. It also matters for the economy. An analysis using American data finds that while improved adherence would increase drug costs it would achieve substantial reductions in overall costs due to fewer complications and hospitalisations [19].

### What can be done?

As this brief review shows, considerable further gains seem possible if people would adhere to medication that they have been prescribed. But how might this be achieved? There is no simple answer. A Cochrane Review found that almost all interventions that were effective in the long-term care were complex [20]. They included combinations of more convenient care, information, reminders, self-monitoring, reinforcement, counselling, family therapy, psychological therapy, crisis intervention, manual telephone follow-up, and supportive care. However, even the most effective interventions did not lead to large improvements in adherence and treatment outcomes and the authors stressed the need for more research. Research that has been undertaken in Israel shows that provision of relevant information on medication [21] and empowerment of nurses can help [22] although, clearly, much more research is needed.

On the other hand, there are some obvious issues that could be addressed. One is cost. There is evidence that chronically ill patients in the United States are reducing their purchases of prescription drugs in the current financial crisis [23]. Concerns have been expressed that the complex system of co-payments for pharmaceuticals in Israel [24] may act as a barrier to adherence [25].

There is also a need to understand better how patients view their medication. Marshall and colleagues reviewed systematically 53 qualitative studies of lay perspectives on hypertension and adherence to treatment, from 16 countries, including Israel [26]. They found a widespread belief hypertension was due mainly to stress and was manifested by symptoms such as headache, dizziness or sweating. Many people believed that, when such symptoms abated, their hypertension was resolved and they discontinue treatment without consulting their position. Many experienced side-effects that they find unpleasant and some feared addiction to treatment. In addition to factors related to their understanding of hypertension and the importance of treatment, there were also a number of external factors, such as the time and cost involved in consulting the physician or obtaining medication, as well as simple forgetfulness. Research among older Israelis specifically identified the problems that arise from multiple medications and where there is lack of trust in the primary caregiver [27]. Finally, there is growing evidence that many of the trials that have evaluated new medications may systematically underestimate the side effects, especially when used in patients with multiple conditions and therapy [28], and who may be quite different from those included in trials. [29]Hence, some of those who do not adhere to prescribed medication may be making rational choices based on their weighing up of the perceived benefits and the often all too apparent side effects.

## Conclusion

In conclusion, the Israeli health system has done well but there is more to be done, in particular by ensuring equitable access to health care, removing financial barriers, and understanding why patients do not take the medication they are prescribed and what can be done to help them do so where it can be established that it will truly benefit them. There is a clear need for more research, beginning with empirical studies of the relative roles of the four factors identified as contributing to cross-national differences in amenable mortality rates and changes over time. This should lead to a fuller analysis of the reasons for Israel's relative position in amenable mortality vis a vis other countries, as well as the reasons for the cross-regional differences within Israel. Turning specifically to adherence to treatment, there is scope for cross-national comparisons of adherence rates and empirical analyses of the causes of differences between countries so that lessons can be learned from those countries with the best adherence rates, including how they have used different policy and programmatic interventions. It is essential that future policies are fully informed by this emerging evidence.

## Competing interests

The authors have no conflicts of interest.

## Authors’ information

Martin McKee (CBE MD DSc FRCP) is Professor of European Public Health at the London School of Hygiene and Tropical Medicine and Director of Research policy at the European Observatory on Health Systems and Policies. With Ellen Nolte he has written extensively on the concept of mortality amenable to medical care. Clara Chow (MBBS, FRACP, PhD) is Consultant Cardiologist at Westmead hospital and Head of the Cardiac Program of research at the George Institute for Global Health, University of Sydney. She has a PhD from the University of Sydney, Australia in cardiovascular epidemiology and international public Health and a Postdoc from McMaster University, Canada. The main focus of her research is cardiovascular disease prevention. Commentary on Nehama Goldberger and Ziona Haklai, Mortality rates in Israel from causes amenable to health care, regional and international comparison.
